# Supplementary material for: Agent-based modeling of nuclear chromosome ensembles identifies determinants of homolog pairing during meiosis
Source: PLoS Comput Biol. 2024 May 13;20(5):e1011416. doi: 10.1371/journal.pcbi.1011416 (PMC11115365; doi:10.1371/journal.pcbi.1011416)
Supplement: S1 Appendix — This Appendix contains additional details and biological discussion regarding the supplemental figures to this manuscript. (PDF) [file pcbi.1011416.s001.pdf]

Supporting Information: Agent-based modeling of nuclear chromosome ensemble identifies determinants of homolog pairing during meiosis

Ariana Chriss[1,2#], G. Valentin Börner[2,3*], Shawn D. Ryan[1,4*],

1 Department of Mathematics and Statistics, Cleveland State University, Cleveland, Ohio, United States of America
2 Department of Biological, Geological, and Environmental Sciences, Cleveland State University, Cleveland, Ohio, United States of America
3 Center for Gene Regulation in Health and Disease, Cleveland State University, Cleveland, Ohio, United States of America
4 Center for Applied Data Analysis and Modeling, Cleveland State University, Cleveland, Ohio, United States of America

# Current Address: Department of Genetics and Genomic Sciences, Icahn School of Medicine, Mount Sinai, New York, New York, United States of America
* Corresponding Authors: g.boerner@csuohio.edu (GVB), s.d.ryan@csuohio.edu (SDR)

Supporting information

Effect of velocity on pairing kinetics

Similar to Fig. 3, we consider the effect of movement velocity on pairing kinetics. We observe that pairing kinetics are accelerated as velocity increases. **Effects of chromosome velocity on pairing kinetics.** (A) Reproduction of Fig 6A, each dot indicates the average pairing levels of all 32 chromosomes of true sizes at the indicated time points. Black (300 nm/s) indicates pairing levels at the velocity of chromosome movements in the model in Fig 3 and Fig 5 Chromosomes fail to pair at velocities around 150 nm/s, likely due to the effect of thermal noise. Increases in 30 nm/s increments increases pairing efficiencies at $t = 9h \sim$ 3-fold, with more modest gains above 240 nm/s where essentially all 16 homologs pair efficiently. (B) Results from S1A Fig were normalized by maximum pairing levels. With increased movement velocities, 50% pairing levels are achieved at progressively earlier time points contains a reproduction of Fig 6A showing how movement velocity changes the homolog pairing efficiency. **Effects of chromosome velocity on pairing kinetics.** (A) Reproduction of Fig 6A, each dot indicates the average pairing levels of all 32 chromosomes of true sizes at the indicated time points. Black (300 nm/s) indicates pairing levels at the velocity of chromosome movements in the model in Fig 3 and Fig 5 Chromosomes fail to pair at velocities around 150 nm/s, likely due to the effect of thermal noise. Increases in 30 nm/s increments increases pairing efficiencies at $t = 9h \sim$ 3-fold, with more modest gains above 240 nm/s where essentially all 16 homologs pair efficiently. (B) Results from S1A Fig were normalized by maximum pairing levels. With increased movement velocities, 50% pairing levels are achieved at progressively earlier time points, rescales each curve in (A) by the maximum pairing levels to obtain insights into pairing kinetics. To investigate this trend's direct effect on pairing kinetics, we compute the distances between homologs as a function of chromosome movement speed. The results are summarized in S2A-D Fig.

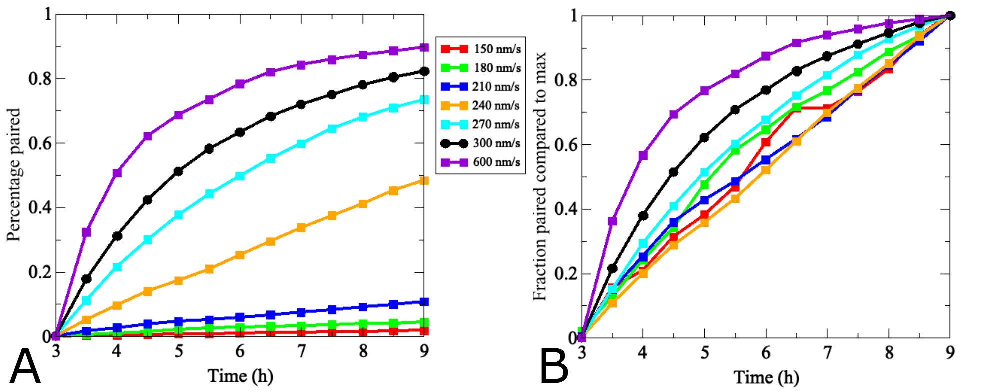

Fig 1. Effects of chromosome velocity on pairing kinetics. (A) Reproduction of Fig 6A, each dot indicates the average pairing levels of all 32 chromosomes of true sizes at the indicated time points. Black (300 nm/s) indicates pairing levels at the velocity of chromosome movements in the model in Fig 3 and Fig 5 Chromosomes fail to pair at velocities around 150 nm/s, likely due to the effect of thermal noise. Increases in 30 nm/s increments increases pairing efficiencies at $t = 9h \sim$ 3-fold, with more modest gains above 240 nm/s where essentially all 16 homologs pair efficiently. (B) Results from S1A Fig were normalized by maximum pairing levels. With increased movement velocities, 50% pairing levels are achieved at progressively earlier time points.

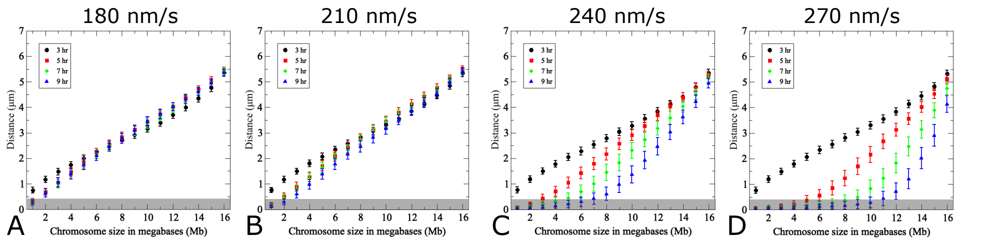

Fig 2. Effects of movement velocity on homologous pairing kinetics. All results for 16 homolog pairs using true chromosome lengths. Chromosomes are arranged according to their initial distance to facilitate comparison with the experimental data set. Chromosome movement velocity is (A) 180 nm/s, (B) 210 nm/s, (C) 240 nm/s, and (D) 270 nm/s. Note the sharp transition between 210 nm/s and 240 nm/s, as also shown in Fig 6A. The pairing distance is highlighted in gray at 400 nm. (200 realizations, error bars indicate SD).

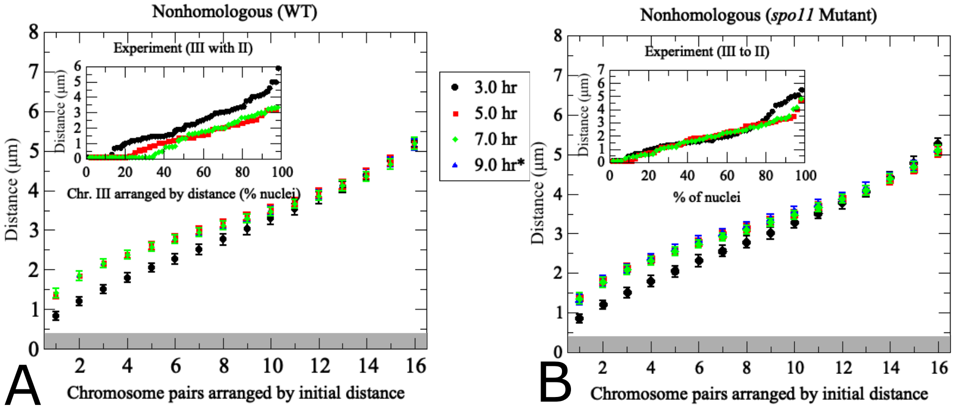

Fig 3. Non-homologous chromosome distances for wild type and *spo11* hypomorph, with experimental observations in the insets. (A) Reproduction of Fig 4B for non-homologous distances in the wild-type simulations. (B) Non-homologous pairs in *spo11* mutants where one of the two homolog partners is matched with a non-homologous chromosome exhibiting an optimally matched initial distance with its cognate homolog partner at $t = 3h$. Inset in (B) shows experimentally determined distances between non-homologous GFP-tagged chromosomes II and III. For details on the experimental conditions see [1]. The pairing distance is highlighted in gray at 400 nm. *Note the experimental data do not include information for $t = 9h$. (200 realizations, error bars indicate SD).

Non-homologous chromosome distances in wild type compared to *spo11* hypomorph

The complementary plot to Fig 7 shows the distribution of non-homologous pairing distances as a function of time. 3 provides a direct comparison between non-homologous chromosome distances in wild type (A) and *spo11* hypomorph (B) where true chromosome lengths are used. Numerically, we tracked the distance between a given homolog and selected the non-homologous chromosome in the same nucleus with the closest initial distance to its homologous pair. The simulation then tracks the dynamics of both throughout time and we plot the distance between a given chromosome and the previously identified non-homologous chromosome. The results indicate that there is no bias in time for a chromosome with any non-homologous chromosome in the nucleus. Also, the results indicate that the mutation does not have a strong effect on non-homologous interactions as it only modifies their strength, but not their qualitative behavior of creating excluded regions. One difference as noted in Fig 7 is that while both the wild type and mutant produce excluded regions, the strength of the repulsion in the wild type makes these regions more severe and drives homologous pairing to occur much faster. This validates the model in that the only different interaction occurs with a given chromosome's homologous mate (compare homologous results in Fig 7 with non-homologous results in 3).

Effect of nucleus size on pairing kinetics

Effect of increase/decrease of interaction forces

Here we consider scenarios where either the attractive force strength C_a dominates 100-fold over the repulsive force strength C_r (S5B Fig) or inversely, the repulsive force strength dominates over the attractive force strength (S5C Fig). Moreover, with the

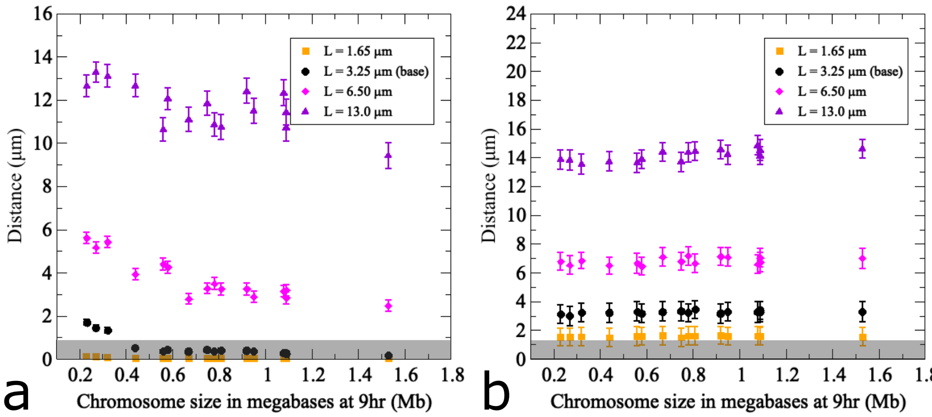

Fig 4. Effects of nucleus size on pairing kinetics and efficiency. Average distances between homolog pairs for actual sizes of yeast chromosomes as a function of nucleus size. Observe that pairing occurs much less frequently as the nucleus radius increases consistent with the roles of confinement and repulsive non-homologous interactions in pairing kinetics. In a high-density environment of a small nucleus, the repulsive interactions have an even larger effect because each chromosome is interacting with many non-homologous neighbors in close proximity. This drives the pairing of homologs by quickly filling the nucleus space with excluded regions due to repulsion. Results over 200 realizations. Error bars indicate SD.

attractive force dominating, pairing of mid-sized and longer chromosomes occurs essentially instantaneously, whereas the process is drawn out over a longer time scale when repulsive forces dominate.

Individual chromosome III dynamics compared to experiment

The experimental data used to calibrate the model were originally reported in [1]. In the experimental setup it was only possible to use GFP tracking to follow homologous distances between chromosome III and itself as well as non-homologous distance between chromosome III and chromosome II. In this supplemental section we present results where the modeling framework developed in this work is used to track only chromosome III for comparison with the experiment rather than all 16 homolog pairs at the same time.

Note that Chromosome III completes pairing in all cells at the last time point in the experiment, whereas it fails to complete pairing in the simulation in the 10% of cells where the two copies of Chromosome III were initially placed at the maximum distance of $> 5\mu$m. The initial distance between homologs may be overestimated in the experiment due to flattening of the cell sphere. Alternatively, the model may fail to consider compensatory mechanisms that ensure pairing of small chromosomes such as Chromosome III (see Fig. 5). Finally, for realizations where Chromosome III pairs were maximally spaced, this may create excluded regions within the nucleus, separating the two copies of Chromosome III from each other, but is accounted for by a large number of realizations so as not to give too much weight to any single observation. Notably, this is not a limitation of the computational model, but rather an expected result when using randomly placed initial conditions.

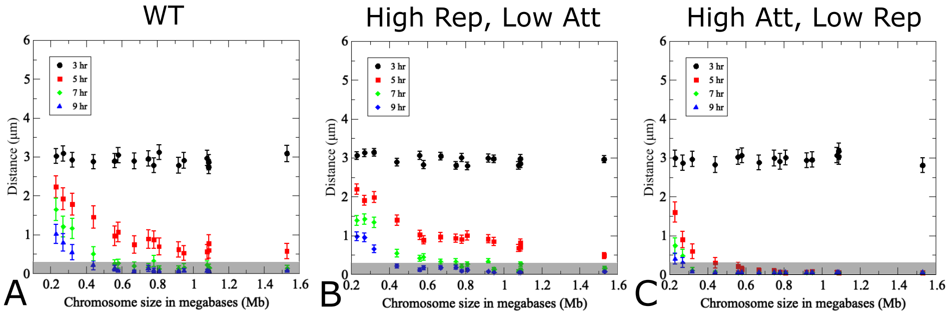

Fig 5. Contributions of attractive and repulsive forces on pairing efficiencies and kinetics. (A) Pairing wild-type model using true chromosome lengths and a standard translational movement velocity of 300 nm/s as primarily studied herein. In (B) the repulsive strength is increased by an order of magnitude $C_r = 0.05$ and the attractive strength is decreased by an order of magnitude $C_a = 0.0005$. In (C) the reverse is true $C_r = 0.0005$ and $C_a = 0.05$ (200 realizations, error bars indicate SD). The pairing distance is highlighted by a gray rectangle.

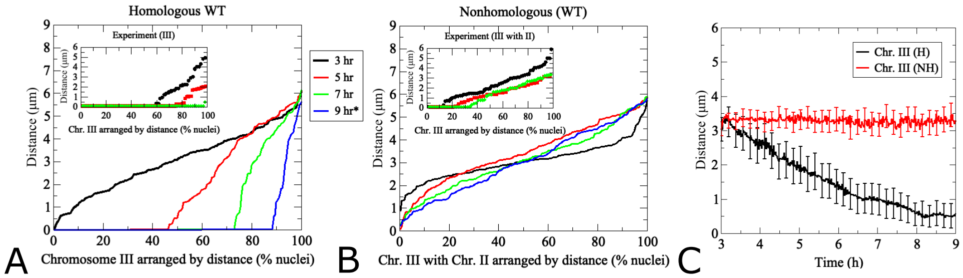

Fig 6. Dynamics of chromosome III. (A) Chromosome III in each of 200 realizations are sorted by their initial distance at $t = 3h$ to their homologous chromosome partner for comparison with experiment. (B) The average distance between Chromosome III and Chromosome II in each of the 200 realizations sorted by their current distance at four time points. (C) Average homologous (Chromosome III with itself) and non-homologous (Chromosome III with Chromosome II) distances as a function of time.

References

1. Sandhu R, Neria FM, Neria JM, Chen X, Hollingsworth NM, Börner GV. DNA helicase $\mathrm{Mph1}^{FANCM}$ ensures meiotic recombination between parental chromosomes by dissociating precocious displacement loops. Developmental Cell. 2020;53(4):458–472.
